# CircLDLR acts as a sponge for miR-667-5p to regulate SIRT1 expression in non-alcoholic fatty liver disease

**DOI:** 10.1186/s12944-022-01740-9

**Published:** 2022-11-29

**Authors:** Xinlu Yuan, Yanyan Li, Song Wen, Chenglin Xu, Congcong Wang, Yanju He, Ligang Zhou

**Affiliations:** Department of Endocrinology and Metabolic Diseases, Shanghai Pudong Hospital, Fudan University Pudong Medical Center, 2800 Gongwei Road, Pudong, Shanghai, 201399 China

**Keywords:** Non-alcoholic fatty liver disease, circRNAs, SIRT1, miR-667-5p, Autophagy

## Abstract

**Background:**

Non-alcoholic fatty liver (NAFLD) is a complex metabolic disease characterized by fatty degeneration of hepatocytes. Circular RNAs (circRNAs) have been reported to be essential for (NAFLD progression. The potential mechanism of circRNA low-density lipoprotein receptor (circLDLR) in the NAFLD was investigated in this study.

**Methods:**

Hepatocyte (Hepa1-6) cells treated with oleic acid/palmitic acid (OA/PA) were used as the in vitro NAFLD model, and C57BL/6 mice fed with high-fat diet (HFD) were used as the in vivo NAFLD model. The circLDLR, LDLR, and miR-667-5p expression were measured by quantitative real-time polymerase chain reaction (qRT-PCR), while the protein levels of Light Chain Microtubule-Associated Protein 3 (LC3) and Sequestosome-1(p62) was examined by western blot. The circLDLR location was confirmed using RNA fluorescence in situ hybridization. Oil red O staining was carried out to measure lipid deposition in cells. The secreted levels of triglyceride (TG) and total cholesterol (TC) were detected through Enzymatic. The existence of the circLDLR/miR-667-5p/sirtuin 1 (SIRT1) regulatory axis was validated by applying the dual-luciferase reporter assay.

**Results:**

The circLDLR expression showed a prominent down-regulation in OA/PA-treated Hepa1-6 cells, whereas the LDLR expression was up-regulated. Overexpression of circLDLR significantly attenuated lipid droplet accumulation in NAFLD models in vitro*/vivo*, reduced TG, TC, and p62 levels, and increased LC3-II levels and the amount of the green fluorescent protein (GFP)-LC3 puncta in cells. CircLDLR and SIRT1 are common targets of miR-667-5p to inhibit the TG and TC and promote the autophagy pathway. SIRT1 knockdown reversed the effects of circLDLR overexpression.

**Conclusions:**

CircLDLR alleviated the development of NAFLD by inducing autophagic flux while modulating the miR-667-5p/SIRT1 axis reversed its effects, suggesting that targeting circLDLR/miR-667-5p/SIRT1 axis may be a promising therapeutic strategy for NAFLD.

## Introduction

It has been reported that non-alcoholic fatty liver disease (NAFLD) is a usual causer for liver disease globally [1], which is primarily triggered by obesity and accompanied by hepatic steatosis [2]. NAFLD is distinguished by the excessive accumulation of triglycerides and lipids in hepatocytes, which leaves the liver more vulnerable to a "second hit" that induces oxidative stress, endoplasmic reticulum stress, and inflammation reaction [3, 4]. Current therapeutic interventions for NAFLD are mainly lifestyle changes and weight loss. Nevertheless, the underlying mechanisms still need more elucidation.

MiRNAs are small noncoding RNAs with around 22 nucleotides, which regulate the target genes expression but do not encode proteins by inducing mRNA degradation and translational repression [5, 6]. Abnormal expression of miRNAs has a close association with NAFLD development and participates in various processes of lipid metabolism [7, 8]. Additionally, several miRNAs have been described to involve in the regulation of hepatic lipid homeostasis, such as miR-34a [9], miRNA-132 [10] and miR-122 [11]. Circular RNAs (circRNAs) are endogenous noncoding RNA molecules, which have become a study hotspot recently for their stable structure, specific tissue and developmental dependence [12, 13]. An earlier study revealed that as a competitive “sponge” for miR-188-3p, circRNA_0048179 could attenuate lipid accumulation in HepG2 cells treated with oleic acid/palmitic acid (OA/PA) [14]. Circ_0057558 can notably improve the progression of NAFLD in vivo via miR-206/Rho-related, coiled-coil containing protein kinase 1 (ROCK1)/ adenosine 5 ‘-monophosphate-activated protein kinase (AMPK) axis [15]. Ample information confirmed that circRNAs regulate the expression of genes via sponging miRNAs or binding to proteins [16]. Research before the present study has also verified that most of the circRNAs were differentially expressed in the NAFLD group [17]. However, the biological functions of circRNA low-density lipoprotein receptor (circLDLR) in NAFLD progression are unknown. Therefore, the exact role of circLDLR and its potential mechanisms in NAFLD still need investigation.

Sirtuin 1 (SIRT1), a member of the sirtuins family, regulates autophagy by the NAD-dependent deacetylation of autophagy-related proteins [18]. SIRT1 is considered to regulate metabolism in different tissues and is involved in lipid metabolism, including fatty acid synthesis, oxidation, and lipogenesis [19, 20]. Accumulating evidence revealed the functional role of SIRT1 in regulating the development and progression of NAFLD [21–23]. This research attempted to explore circLDLR’s role in NAFLD in vivo and in vitro and evaluate whether circLDLR exerts its role in regulating lipid metabolism in NAFLD pathogenesis via targeting miR-667-5p and SIRT1.

## Materials and methods

### Animals

Six-week-old male C57BL/6 mice (Vital River Laboratories, Beijing, China) were acclimatized for 1 week. Mice were housed at constant room temperature (60–65% humidity, 23 ± 2℃) on a cycle with a 12-h dark and 12-h light and accessed to standard chow (10% calories composed of fat) and water freely. The experiments were carried out following a protocol endorsed by the Ethics Committee of Shanghai Pudong Hospital ((2022) N0. (W2-06)).

### Chemical sources

Dulbecco’s modified Eagle’s medium (DMEM; Gibco, Grand Island, NY, USA); oleic acid (OA), palmitic acid (PA), 3-Methyladenine (3-MA), and bovine serum albumin (BSA) were bought from Sigma-Aldric Crop. (St. Louis, MO, USA). The diet with high fat included 20% carbohydrate, 60% fat, and 20% protein (Research Diets Inc, New Brunswick, NJ, USA); while the normal one, 70% carbohydrate, 10% fat, and 20% protein.

### Cell culture and treatment

Hepa1-6 cells (Fuheng Biology, Shanghai, China) were preserved in an expansion medium [DMEM + 10% fetal bovine serum (GIBCO, Carlsbad, CA, USA) + 1% penicillin/streptomycin (GIBCO, Carlsbad, CA, USA)] and placed in a humid incubator (5% CO_2_, 37℃).

Hepa1-6 cells (2 × 10^5^ cells/ml) were seeded into plates at 80% confluence and were then divided into two groups: the oleic acid (OA) + palmitic acid (PA) group and the control group. OA and PA were diluted in 20% and 40% fat-free bovine serum albumin (BSA) from 20 and 40 mM stock solutions, respectively. Subsequently, the media in the culture was replaced with media containing 200 μM OA and 100 μM PA as well as 10% Phosphate Buffered Saline (PBS). After 24 h, 200 μM OA + PA was applied for 24 h to induce fatty acid accumulation on the Hepa1-6 cells, which obtained NAFLD cells. The control group was given the same concentration of fat-free BSA.

### Animal protocols

As previously mentioned, the mouse model of high-fat diet (HFD)-triggered NAFLD was established [17]. After 1 week of acclimatization, a total of 24 mice were randomized into the following three groups (*n* = 6–8), and kept on HFD for further 12 weeks: (1) vector group, tail vein injection of adenovirus [5 × 10^9^ ~ 1 × 10^10^ PFU/mouse]; (2) hsa_circLDLR_0001 overexpression (circLDLR OE) vector group, tail vein injection of 5 × 10^6^ stable hsa_circLDLR_0001 overexpression cells. The adenovirus (Ad)-circLDLR OE and Ad-GFP groups were injected with Ad-circLDLR OE or Ad-GFP through tail veins at an optimum dose of 5 × 10^9^ PFU once a week for 8 weeks; (3) circLDLR OE + 3-MA group, tail vein injection of 5 × 10^6^ stable hsa_circLDLR_0001 overexpression cells and 3-MA (a lysosomal inhibitor) at 30 mg/kg-dose three times per week (MedChemExpress, NJ, USA). Mice were sacrificed under anesthesia with 50 mg/kg pentobarbital sodium intraperitoneally after the last injection. One part of fresh liver tissues was used for Oil Red O (ORO) staining, and the other portion was maintained at -80 °C and applied for western blot and quantitative real-time polymerase chain reaction (qRT-PCR) assays.

In addition, to further verify that miR-667-5p could down-regulate SIRT1 expression, we divided 6 NAFLD mice into two groups: (1) 0.5 μg miR-667-5p mimics dissolved in jetPEI vehicle (Polyplus Transfection, Illkirch, France); (2) 0.5 μg NC mimics dissolved in jetPEI vehicle. The mice were injected via tail vein 3 times a week for 4 weeks. After the last injection, mice were sacrificed under anesthesia with an intraperitoneal injection of 50 mg/kg sodium pentobarbital. Fresh liver tissue was stored at -80 °C for Western blot.

### RNase R and Actinomycin D treatments

The circLDLR stability was detected by performing RNase R and Actinomycin D assays. Total RNA (10 μg) of lysed Hepa1-6 cells was treated with 40 U RNase R (Solarbio, Beijing, China) at 37 °C for 15 min. RNase-free total RNA was utilized to be a control. The circLDLR and LDLR levels were then determined using qRT-PCR.

Hepa1-6 cells (5 × 10^4^ cells/well) were seeded on 6-well plates and treated with Actinomycin D (Abcam, Cambridge, MA, USA) for 0, 8, 16 and 24 h. After that, the circLDLR and LDLR expression was detected via qRT-PCR.

### RNA-fluorescence in situ hybridization (FISH)

The hsa_circLDLR_0001 location in Hepa1-6 cells was observed by conducting the FISH assay. Briefly, cells were digested in proteinase K (Servicebio, Wuhan, Hubei, China) for 6 min after fixation in 4% paraformaldehyde (DEPC). After rinsing with PBS, the pre-hybridization solution was dripped for 1 h. Subsequently, the hybridization solution containing the circLDLR probe (500 nM) was added and cultured overnight at 42 °C, followed by the addition of a hybridization solution containing the two-labeled probe (1:400 dilution) and incubation for 3 h. After being blocked with rabbit serum, the cells were cultured using mouse anti-DIG-HRP (Jackson, Lancaster, PA, USA) and FITC-TSA. Next, the location of hsa_circLDLR_0001 was determined by staining the nucleus with DAPI (Servicebio, Wuhan, Hubei, China) and viewing the results using a fluorescent microscope (Nikon, Tokyo, Japan).

### Cell transfection

Hsa_circLDLR_0001 full length was cloned into the overexpression vector (pcDNA3.1). miR-667-5p mimics, negative control (NC) mimics, NC siRNA, SIRT1 siRNA, and SIRT1 overexpression (SIRT1 OE) were synthesized and bought from Genepharma (Shanghai, China). Thereafter, plasmids were transfected into Hepa1-6 cells treated with OA and PA by applying Lipofectamine 3000 Transfection Reagent (Invitrogen, Carlsbad, CA, USA) following the recommendations of the manufacturer. Then, transfected cells were collected for other experiments.

### Autophagy flux assay using fluorescent-tagged light chain microtubule-associated protein 3 (LC3)

Overexpressed hsa_circLDLR_0001 NAFLD cells were transfected with Ad-LC3-GFP-Red-Fluorescent Protein (RFP) adenovirus (Asia-Vector Biotechnology, Shanghai, China) that displays a specific marker for autophagosome formation for autophagy detection, followed the instructions of the manufacturer. Next, PBS was used to wash cells before fixing them for 30 min with 4% paraformaldehyde, followed by blocking using block solution (Beyotime, Shanghai, China). Subsequently, cell nuclei were stained using DAPI (1:10,000 dilution; Beyotime, Shanghai, China) and the images were captured by confocal laser scanning microscopy (Olympus Corporation, Tokyo, Japan). Five different fields of view were randomly acquired from each section and the GFP- and RFP-LC3 puncta were counted.

### Transmission electron microscopic (TEM) analysis

The liver fragments were first fixed with an electron microscope fixative (Servicebio, Wuhan, Hubei, China) at 4 °C for 2–4 h. Next, a further fixation using 1% osmium tetroxide in 0.1 M PBS buffer at 20 °C for 2 h was performed. Subsequently, the fixed tissues were embedded in epon after being dehydrated using a graded ethanol series. The images were obtained under a transmission electron microscope (HITACHI, Beijing, China) after staining ultrathin sections (60 nm) with lead citrate and uranyl acetate.

### Dual-luciferase reporter assays

The luciferase reporter vectors circLDLR-Wt, circLDLR-Mut, SIRT1-Wt, and SIRT1-Mut were created via inserting wild-type (Wt) or mutant (Mut) sequences of circLDLR or SIRT1 3ʹUTR with the putative miR_668_5p binding sites into the pmirGLO plasmid (Promega, Madison, WI, USA). Subsequently, miR-NC or miR-668-5p mimics with the matching vector was used to transfect the cells for 48 h. The Dual-luciferase Reporter® Assay System (Promega, Madison, WI, USA) was carried out to examine the activity of luciferase.

### Quantitative real-time polymerase chain reaction (qRT-PCR)

TRIzol reagent (Invitrogen, Carlsbad, CA, USA) was used to separate total RNAs from Hepa1-6 cells and liver tissues, and the PrimeScript™ RT reagent kit (TransGen Biotech, Beijing, China) was applied to reverse-transcribed the RNAs into cDNA. Next, the qPCR was performed via ABI Stepone plus fluorescence quantitative PCR instrument (illumina, San Diego, CA, USA). Analysis of the relevant miRNA and mRNA expression levels was conducted by the 2^–ΔΔCt^ method. The glyceraldehyde 3-phosphate dehydrogenase (GAPDH) and U6 small nuclear RNA (snRNA) functioned as internal reference. Primers employed in this study were shown in Table 1. The thermal cycling conditions were 96 °C for 5 min, and then 40 cycles of 95 °C for 30 s and 68 °C for 20 s.

**Table 1:** Primer sequences for qRT-PCR

Gene	Primers
circLDLR	F: 5’ – TGGCCATCTATGAGCTTCATGT
	R: 5’ – GCCACTGGATGTTTTCGGTC
LDLR	F: 5’ – GCCACATGGTATGAGGTTCC
	R: 5’ – GCTCGTCCTCTGTGGTCTTC
GAPDH	F: 5’ – AGGTCGGTGTGAACGGATTTG
	R: 5’ – GGGGTCGTTGATGGCAACA
miR-667-5p	5’ –GCCGAGCGGUGCTGGTGGAG-3’
miR-492-3p	5’ –GCCGAGTGAAGGTCCTACTG-3’
miR-676-3p	5’ –GCCGAGCCGUCCTGAGGTTG-3’
miR-702-3p	5’ –GCCGAGTGCCCACCCTTTA-3’
miR-770-3p	5’ –GCCGAGCGTGGGCCTGACGT-3’
miR-1188-5p	5’ –CTCAACTGGTGTCGTGGA-3’
U6	F: 5’-CTCGCTTCGGCAGCACA-3’
	R: 5’-AACGCTTCACGAATTTGCGT-3’

*Abbreviations*: *F* Forward, *R* Reverse, *circLDLR* circular RNA low-density lipoprotein receptor, *GAPDH* glyceraldehyde 3-phosphate dehydrogenase

### Oil red O (ORO) staining

Liver tissues and cells of the mice were harvested and fixed using 4% paraformaldehyde (Sangon Biotech, Shanghai, China) and stained with ORO solution (Sigma-Aldric Crop., St. Louis, MO, USA) to detect intracellular oil droplets upon microscopic analysis, as described previously [24]. Images were captured under a microscope. Then, the ORO working solution was eluted with 60% isopropanol (Sinopharm, Shanghai, China) and the absorbance was quantified at 510 nm. After that, the slides were observed in 200 × and 400 × scope.

### Measurement of triglyceride and total cholesterol

The triglyceride (TG) and total cholesterol (TC) contents of Hepa1-6 cells and liver tissues were analyzed using enzymatic TG and TC Assay kits (Nanjing Jiancheng Bioengineering Institute, Nanjing, Jiangsu, China) following the directions of the manufacturer. Briefly, Hepa1-6 cells were pretreated with or without OA/PA and lysed, then the supernatant was taken for TG and TC quantification. Additionally, homogenized the liver tissues and lysed them. Next, the supernatant was taken after centrifugation for subsequent TG and TC quantification. Total protein levels in each sample were used to normalize the TG and TC contents.

### Western blotting analysis

After lysis with RIPA lysis buffer (Beyotime, Shanghai, China), the Hepa1-6 cells and liver tissue were centrifuged at 14,000 × g at 4 °C for 15 min. Then, 10% sodium dodecyl sulfate–polyAcrylamide gel electrophoresis (SDS-PAGE; Sinopharm, Shanghai, China) was used to resolve extracted proteins and polyvinylidene difluoride (PVDF) membranes (Millipore, Shanghai, China) was used to blot them. The membrane was blocked with 5% skimmed milk in Tris-buffered saline (room temperature, 1 h), followed by culturing with primary antibodies against LC3 (Cell Signaling Technology, Danvers, MA, USA; 1:1000 dilution), Sequestosome-1(p62) (Cell Signaling Technology, Danvers, MA, USA; 1:3000), LAMP2 (Abcam, Cambridge, MA, USA; 1:1000), mTOR (Cell Signaling Technology, Danvers, MA, USA; 1:1000) and SIRT1 (Abcam, Cambridge, MA, USA; 1:1000) at 4 °C overnight. Next, HRP-Goat anti-rabbit secondary antibodies (ASPEN, Wuhan, Hubei, China; 1:10,000) were cultured with it for 2 h at room temperature. GAPDH (Abcam, Cambridge, MA, USA; 1:10,000) was applied as a loading control. Chemiluminescence (ECL) substrate (Thermo Fisher Scientific, Waltham, MA, USA) was used to see the protein bands and the ImageJ software was utilized to semi-quantify them.

### Bioinformatics analysis

The effects of hsa_circLDLR_0001 on miR_667_5p or SIRT1 were analyzed by CircInteractome (https://omictools.com/circinteractome-tool) and TargetScan database (https://www.targetscan.org/), and then the collection of hsa_circLDLR_0001 targeting miRNAs was constructed. Furthermore, the experiment-proven miRNAs induced by hepatic steatosis were gathered to build up another miRNA set [6].

### Statistical analysis

Data were expressed as the mean ± standard deviation (SD). PRISM® GraphPad 8.0 software, Tukey’s post-hoc tests, and one- or two-way analysis of variance (ANOVA) were conducted to analyze all data. A minimum of three experimental replicates were assessed for each sample. *P* < 0.05 was considered a prominent level, and the corresponding *p*-values were indicated.

## Results

### OA/PA induces circLDLR downregulation and lipogenesis in hepatocytes

**Fig. 1:**
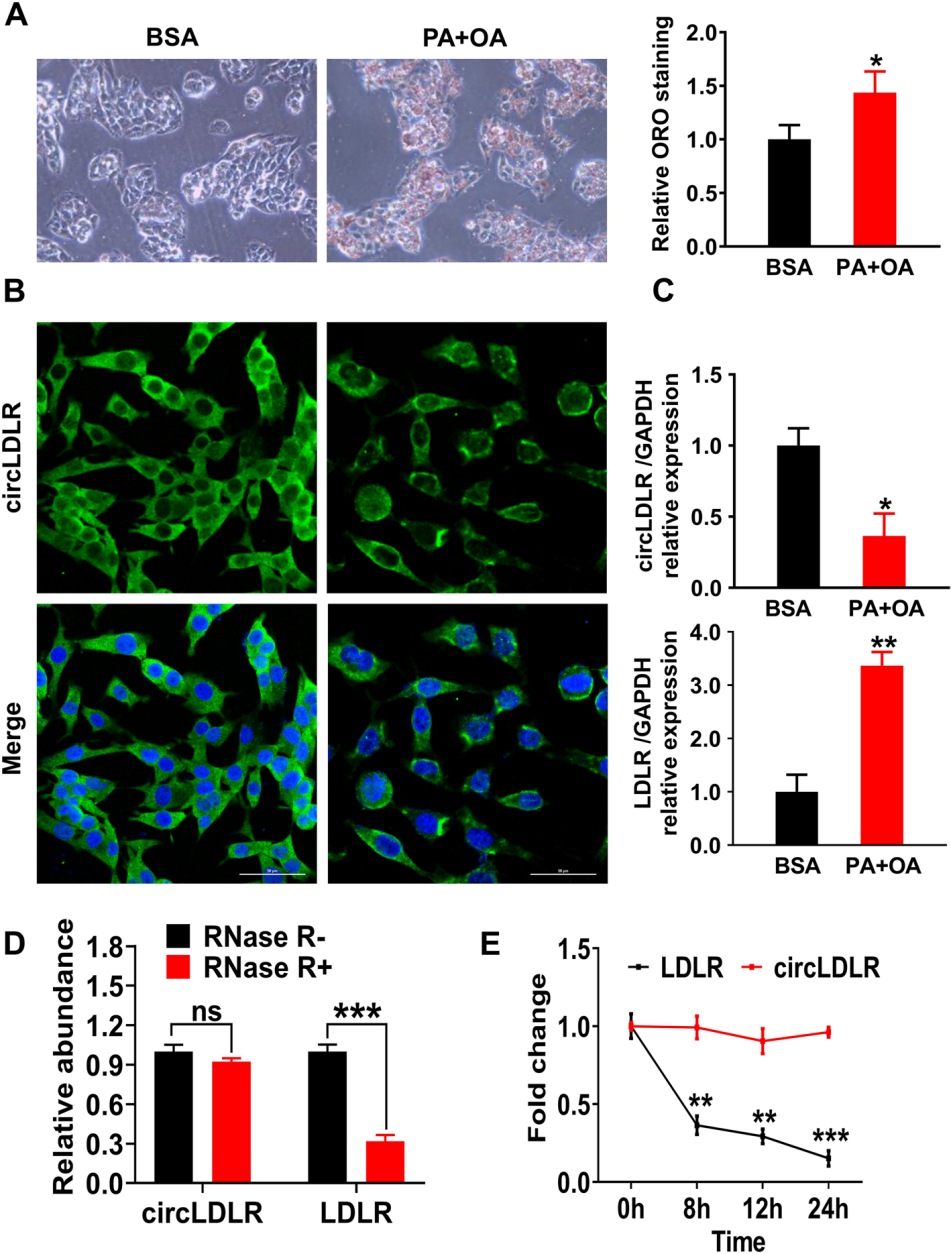
OA/PA induces circLDLR downregulation and lipogenesis in hepatocytes. **A** ORO staining showed the lipid droplets in Hepa1-6 cells. ^*^P < 0.05 *vs.* BSA group. **B** FISH showed the localization of circLDLR in Hepa1-6 cells. The nuclei were stained using DAPI solution. **C** The relative expression of circLDLR and LDLR in Hepa1-6 cells was measured via qRT-PCR. ^*^P < 0.05, ^**^P < 0.01 *vs.* BSA group. **D** The relative circLDLR expression and its linear LDLR with and without RNase R treatment by qRT-PCR in Hepa1-6 cells. ^***^P < 0.001 *vs.* RNase R- group. **E** The LDLR and circLDLR abundance in Actinomycin D-treated Hepa1-6 cells at specific time points was evaluated using qRT-PCR. ^**^P < 0.01, ^***^P < 0.001 *vs.* circLDLR group

To study the circLDLR expression in NAFLD, a hepatic steatosis model was built up with OA/PA-treated Hepa1-6 cells. ORO staining demonstrated that the intracellular lipids were significantly deposited after treatment with OA and PA (Fig. 1A). Furthermore, the FISH analysis demonstrated that circLDLR was primarily concentrated on the cytoplasm (Fig. 1B). qRT-PCR indicated that the OA + PA group had markedly lower circLDLR expression level than the BSA group (*P* < 0.05), while LDLR expression was notably upregulated (*P* < 0.01, Fig. 1C). Afterwards, RNase R and Actinomycin D were conducted to explore circLDLR’s stability. According to the results, circLDLR had more resistance to RNase R digestion than the linear LDLR (*P* < 0.001, Fig. 1D) and was more stable in Hepa1-6 cells under Actinomycin D treatment (Fig. 1E).

### In vitro overexpression of circLDLR promotes activation of hepatic autophagy

**Fig. 2:**
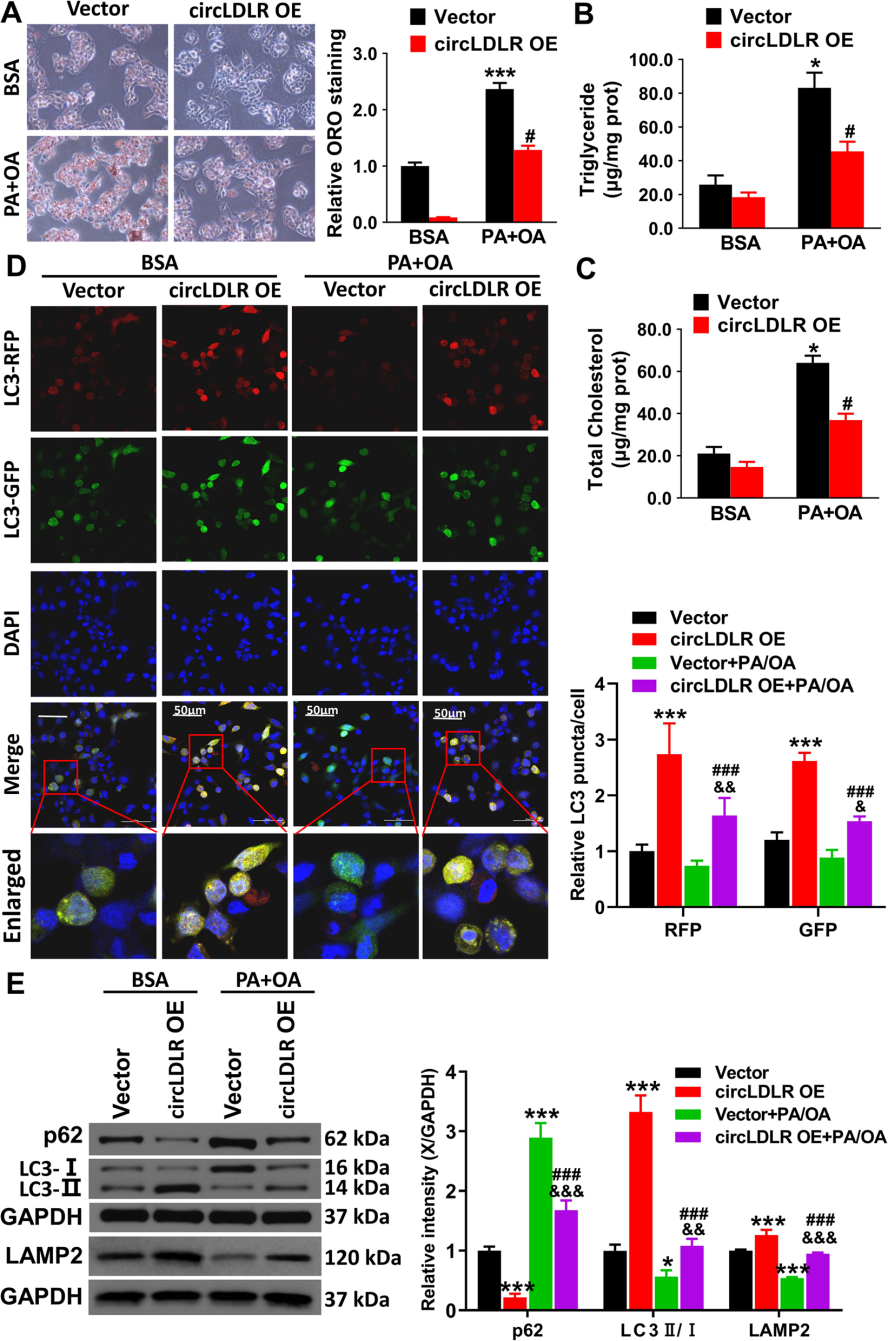
In vitro overexpression of circLDLR ameliorates the development of NAFLD by activating the autophagy signaling pathway. **A** ORO staining showed the lipid droplets in overexpressed circLDLR Hepa1-6 cells. **B** and **C** Hepa1-6 cells with or without OA/PA treatment to transfect with circLDLR overexpression plasmid, and an enzymatic method was used to measure intracellular TG and TC contents. *P < 0.05 *vs.* BSA group, ^#^*P* < 0.05 *vs.* vector group. **D** Hepa1-6 cells with overexpressed circLDLR were transfected with Ad-LC3-GFP-RFP adenovirus and then assessed by immunofluorescence using confocal microscopy. The number of RFP- and GFP-LC3 puncta per cell were quantified and the amount of dots was calculated in the minimum of three independent visual fields from three separate wells. ^*^P < 0.05, ^***^P < 0.001 *vs.* vector group; ^##^*P* < 0.01, ^###^*P* < 0.001 *vs.* circLDLR OE group; &&*P* < 0.01 *vs.* vector + PA/OA group. **E** LC3 and p62, and LAMP2 expressions in Hepa1-6 cells were determined using western blot and relative quantification. ^*^P < 0.05, ^**^P < 0.01, ^***^P < 0.001 *vs.* vector group; ^###^*P* < 0.001 *vs.* circLDLR OE group; ^&&^*P* < 0.01, ^&&&^*P* < 0.001 *vs.* vector + PA/OA group

Autophagy can affect the development and progression of NAFLD by regulating lipid metabolism in hepatocytes and inflammatory response in liver tissue, so we examined whether circLDLR overexpression leads to the activation of autophagy in Hepa1-6 cells. Firstly, ORO staining results showed that circLDLR overexpression alleviated lipid droplet aggregation in OA/PA-pretreatment cells (Fig. 2A). Moreover, TG and TC, the routine index in blood lipid tests, could accurately determine the degree of hepatic steatosis. Biochemical assay results suggested that the TG (Fig. 2B) and TC (Fig. 2C) contents were significantly higher in OA/PA pretreated Hepa1-6 cells than that in the BSA group (*P* < 0.05), and circLDLR overexpression plasmid transfection prominently reduced their contents (*P* < 0.05). In addition, this study explored the effects of overexpressed circLDLR on autophagic flux (Fig. 2D). The rationale of the assay is based on the pH difference between acidic and neutral autophagosomes and the difference in pH sensitivity exhibited by GFP and RFP to monitor the progression from autophagosomes to autolysosomes [25]. The strength of the autophagic flow can be clearly seen by the counting of different colored spots. RFP is used to label and track LC3, and the attenuation of GFP indicates the fusion of lysosomes with autophagic vesicles to form autophagic lysosomes. Based on the red/green fluorescence co-localization, the bright yellow fluorescent spots after Merge are the autophagosomes. Co-incubation of overexpressed circLDLR NAFLD or overexpressed circLDLR Hepa1-6 cells with adenovirus dramatically promoted the accumulation of GFP- and RFP-LC3 (*P* < 0.001), indicating that circLDLR overexpression induced autophagy. However, the circLDLR OE + PA/OA group had an obvious lower accumulation of GFP- and RFP-LC3 than the circLDLR OE group (*P* < 0.05, *P* < 0.01), which demonstrated that OA/PA pretreatment also had a certain inhibitory effect on autophagy flux, but the role of circLDLR and autophagy on NAFLD is still unclear. Similarly, LC3-II levels were markedly elevated in cells treated with overexpressed circLDLR and overexpressed circLDLR NAFLD cells in comparison to cells treated with the vector (*P* < 0.001, Fig. 2E). It has been suggested that p62 levels are correlated with autophagic degradation [26]. This study found that circLDLR overexpression dramatically reduced the level of p62 in the circLDLR OE + PA/OA group by comparison with the vector + PA/OA group (*P* < 0.001, Fig. 2E). The lysosomal marker LAMP2 is often used to track the progression of autophagy. CircLDLR overexpression leads to LAMP2 upregulation in PA/OA-treated Hepa1-6 cells ((*P* < 0.001, Fig. 2E). Therefore, circLDLR overexpression was speculated to inhibit the development of NAFLD by inducing autophagic flux and promoting p62 protein degradation.

### In vivo overexpression of circLDLR attenuates lipid accumulation in liver tissue and activates autophagy signaling pathway

**Fig. 3:**
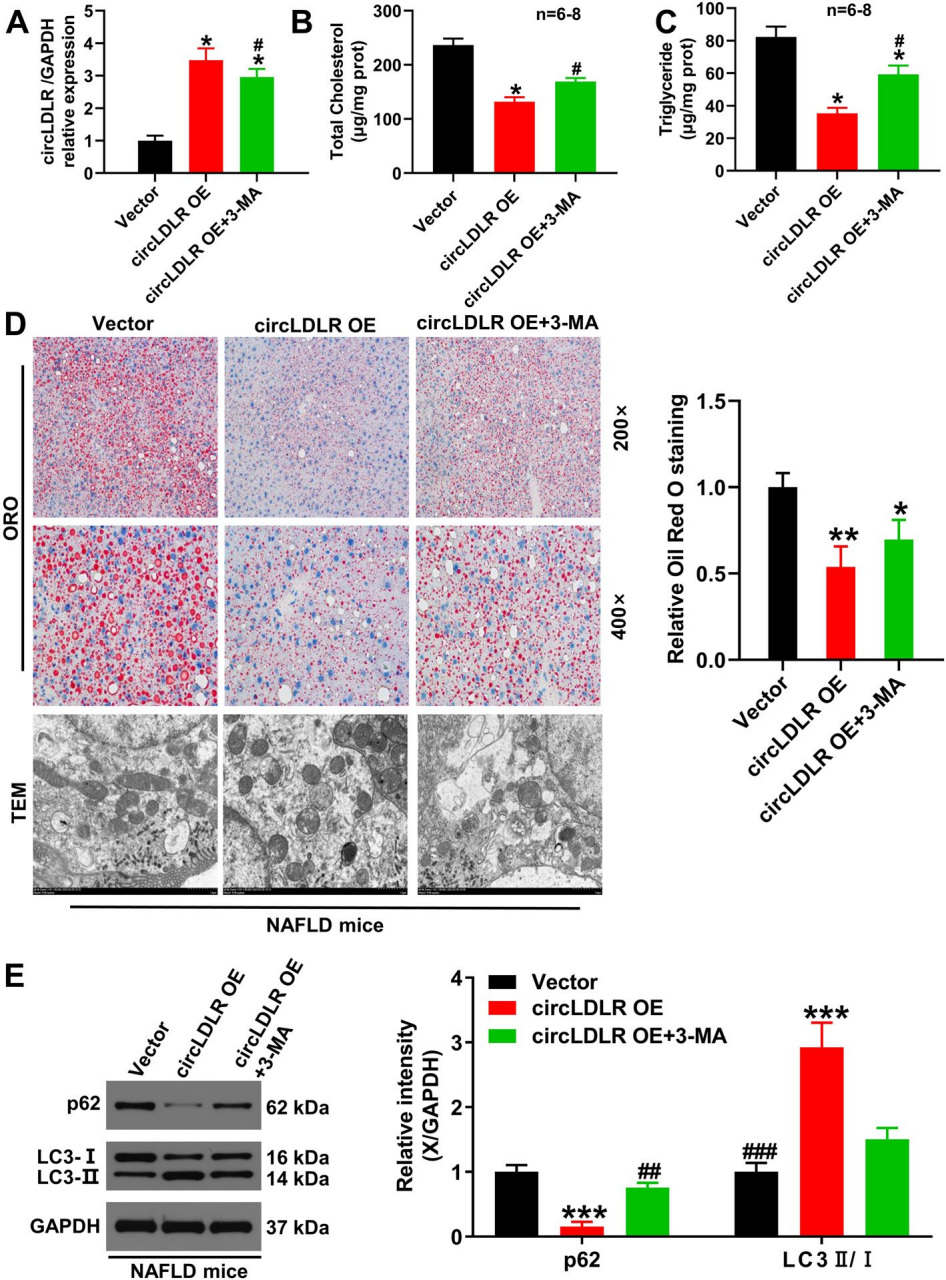
In vivo overexpression of circLDLR alleviates the NAFLD development through the autophagy signaling pathway activation**.** HFD-induced mice were treated with circLDLR overexpression plasmid and then injected with or without 3-MA, **A** the circLDLR expression in liver tissues of NAFLD mice was quantified via qRT-PCR. ^*^P < 0.05 *vs.* Vector group; ^#^*P* < 0.05 *vs.* circLDLR OE group. **B** and **C** An enzymatic method was performed to measure the contents of TG and TC from liver tissues. ^*^P < 0.05 *vs.* Vector group, ^#^*P* < 0.05 *vs.* circLDLR OE group. **D** The effects of in vivo overexpression of circLDLR were investigated through ORO staining and TEM analysis on the liver histology of NAFLD mice. ^*^P < 0.05, ^**^P < 0.01 *vs.* Vector group. **E** LC3 and p62 expressions in the livers of NAFLD mice were examined using western blot and relative quantification. ^***^P < 0.001 *vs*. Vector group; ^##^*P* < 0.01, ^###^*P* < 0.001 *vs.* circLDLR OE group

To evaluate the effect of circLDLR on lipid accumulation and autophagic signaling pathways in liver tissue, the HFD-induced NAFLD mouse models were separated into the following groups: vector group, circLDLR OE group, and circLDLR OE + 3-MA group. Figure 3A showed that the circLDLR expression level was markedly raised in the circLDLR OE in comparison to the vector group (*P* < 0.05). However, by comparison with the circLDLR OE group (*P* < 0.05), it significantly declined in the circLDLR OE + 3-MA group. Additionally, in vivo overexpression of circLDLR notably diminished the TG and TC contents in HFD-induced liver tissue (*P* < 0.05, Fig. 3B and C), whereas 3-MA significantly reversed this result (*P* < 0.05). Oil red O staining results revealed that NAFLD mice injected with circLDLR OE exhibited smaller and fewer lipid droplets in liver sections, however, the addition of 3-MA increased the size and number of lipid droplets (Fig. 3D). Furthermore, TEM images showed that double-membrane autophagic vacuoles accumulated in circLDLR-overexpressing liver tissue (Fig. 3D). In line with in vitro results, the overexpression of circLDLR prominently increased the LC3II levels, whereas p62 was significantly degraded in vivo (*P* < 0.001, Fig. 3E). Nevertheless, the above results were reversed by the autophagy inhibitor, 3-MA (*P* < 0.01, *P* < 0.001), thus suggesting that in vivo circLDLR overexpression may induce autophagy to attenuate the development of NAFLD.

### CircLDLR serves as an efficient miR-667-5p sponge in an in vitro model of NAFLD

**Fig. 4:**
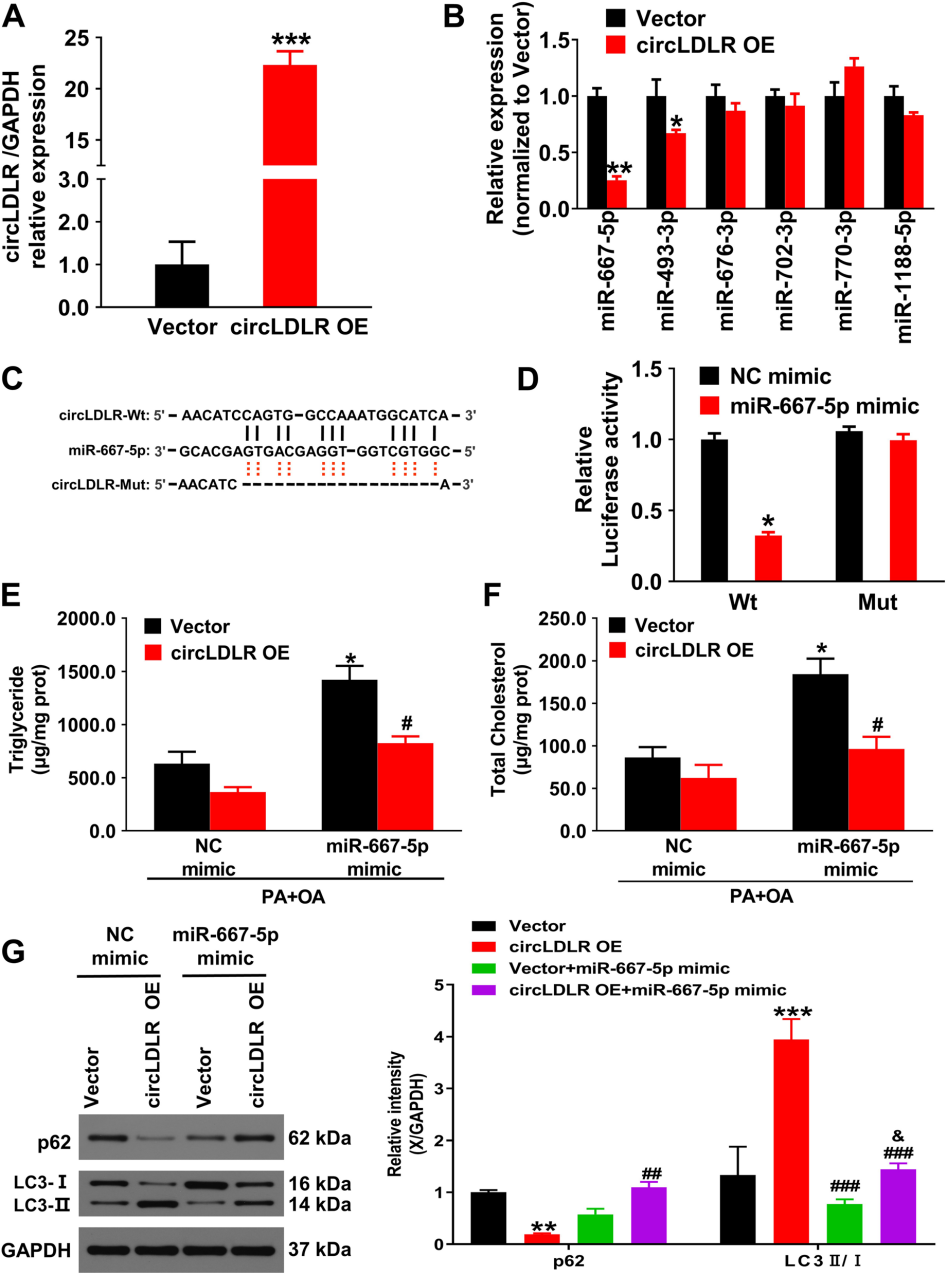
CircLDLR competition combined with miR-667-5p affects the autophagy pathway. **A** The circLDLR expression level in Hepa1-6 cells was measured via qRT-PCR. **P < 0.01 *vs.* vector group. **B** The expression of the top six candidate miRNAs identified by the CircNet database was measured via qRT-PCR in Hepa1-6 cells. ^*^P < 0.05, ^**^P < 0.01 *vs.* vector group. **C** The underlying binding sequences between circLDLR and miR-667-5p were predicted by TargetScan. **D** The binding association of miR-667-5p with circLDLR was validated through a Dual-luciferase assay. ^*^P < 0.05 *vs.* NC mimic. **E** and **F** The TG and TC contents in Hepa1-6 cells were measured through enzymatic method. ^*^P < 0.05 *vs.* NC mimic group; ^*#*^*P* < 0.05 *vs.* vector group. **G** LC3 and p62 expressions in Hepa1-6 cells were measured through western blot and relative quantification. ^**^P < 0.01, ^***^P < 0.001 *vs.* vector group; ^##^*P* < 0.01, ^###^*P* < 0.001 *vs.* circLDLR OE group; ^&^*P* < 0.05 *vs.* vector + miR-667-5p group

To analyze the circLDLR’s underlying mechanism in regulating NAFLD progression, this study selected the top six (miR-667-5p, miR-493-3p, miR-676-3p, miR-702-3p, miR-770-3p, miR-1188-5p) candidate miRNAs based on the bioinformatics analysis results [17]. Firstly, qRT-PCR showed that overexpression of circLDLR in vitro remarkably elevated the circLDLR expression level (*P* < 0.01, Fig. 4A). Next, this paper detected that miR-667-5p and miR-493-3p levels were significantly downregulated in Hepa1-6 cells via qRT-PCR (Fig. 4B), with the miR-667-5p showing the most notable downregulation (*P* < 0.01), so miR-667-5p was selected as the study object. Figure 4C showed the predicted binding sites between miR-667-5p and circLDLR. Then, to further validate the hypothesis, a dual-luciferase reporter assay was applied. In comparison to the group with transfection of circLDLR-WT and mimic NC, the one with transfection of circLDLR-WT and miR-667-5p mimic (*P* < 0.05) had prominently weaker luciferase activity. However, the mut groups showed no noticeable difference (Fig. 4D). The results indicate that miR-667-5p directly binds to circLDLR.

Next, this study determined whether circLDLR inhibits NAFLD progression by interacting with miR-667-5p. Figure 4E and F showed that the TG and TC levels in Hepa1-6 cells dramatically rose after the miR-667-5p mimic in comparison to the NC mimic group (*P* < 0.05). Moreover, western blot demonstrated that miR-667-5p mimic significantly inhibited the increase of LC3-II level and promoted the p62 expression level in Hepa1-6 cells (Fig. 4G). Therefore, circLDLR was speculated to enhance autophagy and its related indicators, while miR-667-5p can reverse its effect, indicating that the effect of cicrLDLR on autophagy relies on miR-667-5p.

### MiR-667-5p can target SIRT1 to regulate autophagy signaling pathway

**Fig. 5:**
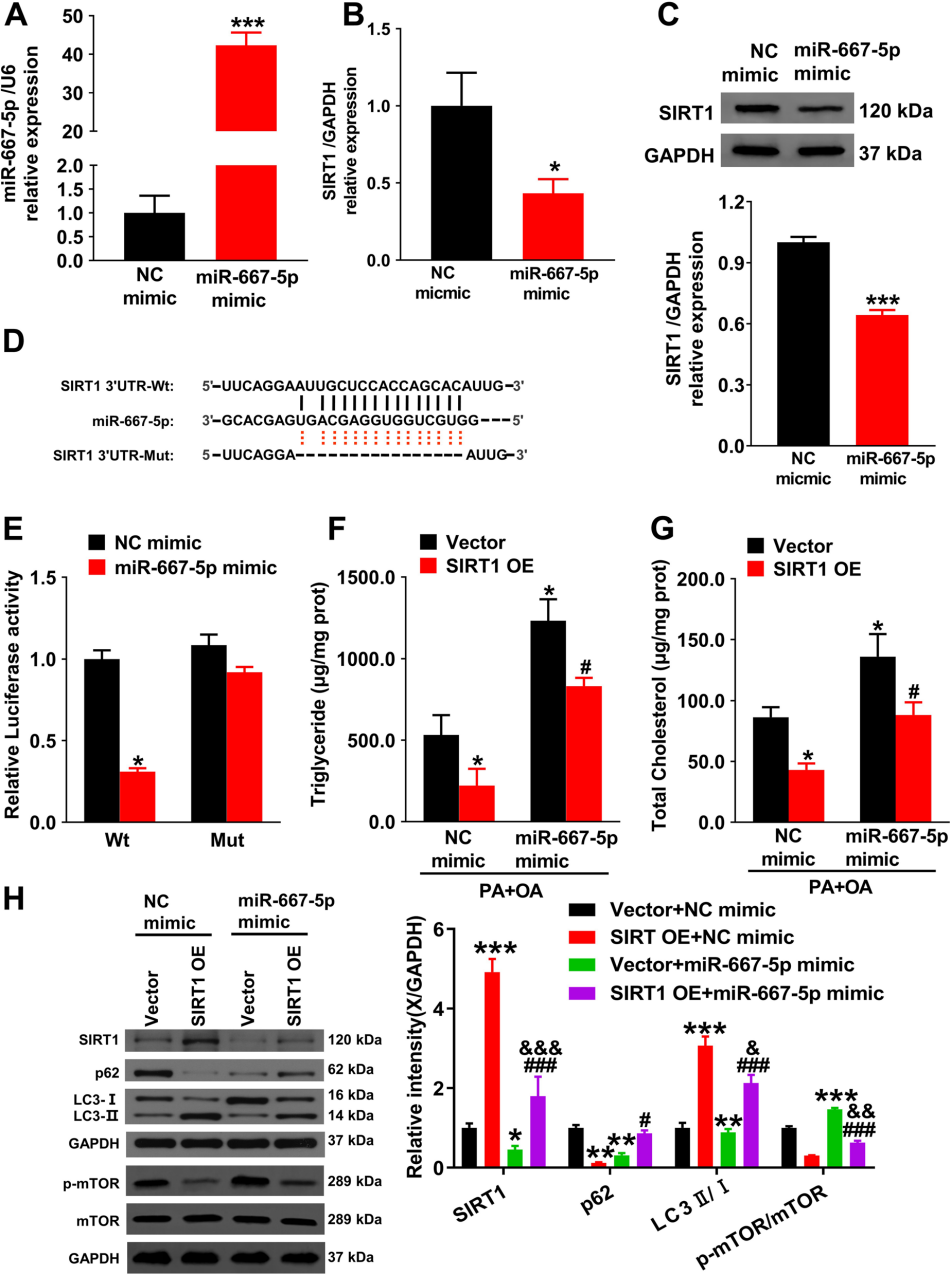
miR-667-5p can target SIRT1 to regulate the autophagy signaling pathway. **A** qRT-PCR detection of miR-667-5p level in Hepa1-6 cells. ***P < 0.001 *vs.* NC mimic group. **B** qRT-PCR detection of SIRT1 mRNA expression in Hepa1-6 cells. ^*^P < 0.05 *vs.* NC mimic group. **C** SIRT1 levels in NAFLD mice were detected by western blot. ^***^P < 0.001 *vs.* NC mimic group. **D** The potential binding sequences between circLDLR and SIRT1 were predicted by TargetScan. **E** The binding association of miR-667-5p with SIRT1 was verified by determining the luciferase activity. ^*^P < 0.05 *vs.* WT + NC mimic group. **F** and **G** The TG and TC contents in Hepa1-6 cells were measured via enzymatic method. ^*^P < 0.05 *vs.* vector + NC mimic group; ^#^*P* < 0.05 *vs.* vector + miR-667-5p mimic group. **H** SIRT1, p62, LC3, and mTOR expressions in Hepa1-6 cells were determined using western blot and relative quantification by densitometry. ^*^P < 0.05, ^**^P < 0.01, ^***^P < 0.001 *vs.* vector + NC mimic group; ^#^*P* < 0.05, ^###^*P* < 0.001 *vs.* SIRT1 OE + NC mimic group; ^&^*P* < 0.05, ^&&&^*P* < 0.001 *vs.* vector + miR-667-5p mimic group

To further examine the miR-667-5p's potential mechanism in regulating the autophagy pathway, miR-667-5p was transfected into Hepa1-6 cells. qRT-PCR suggested that the miR-667-5p expression level prominently up-regulated (*P* < 0.001, Fig. 5A). However, miR-667-5p mimic significantly reduced the SIRT1 mRNA expression by comparison with the NC mimic group (*P* < 0.05, Fig. 5B), indicating that miR-667-5p has a negative feedback regulation on SIRT1. Subsequently, we transfected miR-667-5p in NAFLD mice and further confirmed this result by western blot (Fig. 5C). To validate the miR-667b-3p binding sites on SIRT1 mRNA (Fig. 5D), the study applied the dual-luciferase reporter assay (Fig. 5E). The group which transfected with SIRT1-WT and miR-667-5p mimic had dramatically diminished the activity of luciferase in comparison to one transfected with SIRT1-WT and mimic NC (*P* < 0.05). However, the mut groups showed no prominent difference. The results pointed out that miR-667-5p directly binds to SIRT1.

Then, this study investigated whether miR-667-5p regulates the autophagy signaling pathway via interacting with SIRT1. In the PA + OA-induced in vitro NAFLD model, miR-667-5p could promote the levels of TG (Fig. 5F) and TC (Fig. 5G) in Hepa1-6 cells, which were reversed by SIRT1 overexpression (*P* < 0.05). The phosphorylation level of mTOR, a major downstream regulator of SIRT1, was significantly inhibited, corroborating the overexpression of SIRT1. Additionally, western blotting analysis demonstrated that miR-667-5p significantly inhibited LC3-II protein levels in the SIRT overexpression group. At the same time, the p62 protein expression level was markedly raised in the SIRT1 OE + miR-667-5p group in comparison to the vector + miR-667-5p mimic group (Fig. 5H). Therefore, this study believes that miR-667-5p can interact with SIRT1 to regulate the autophagy signaling pathway.

### circLDLR regulating SIRT1-autophagy signaling pathway to alleviate NAFLD progression

**Fig. 6:**
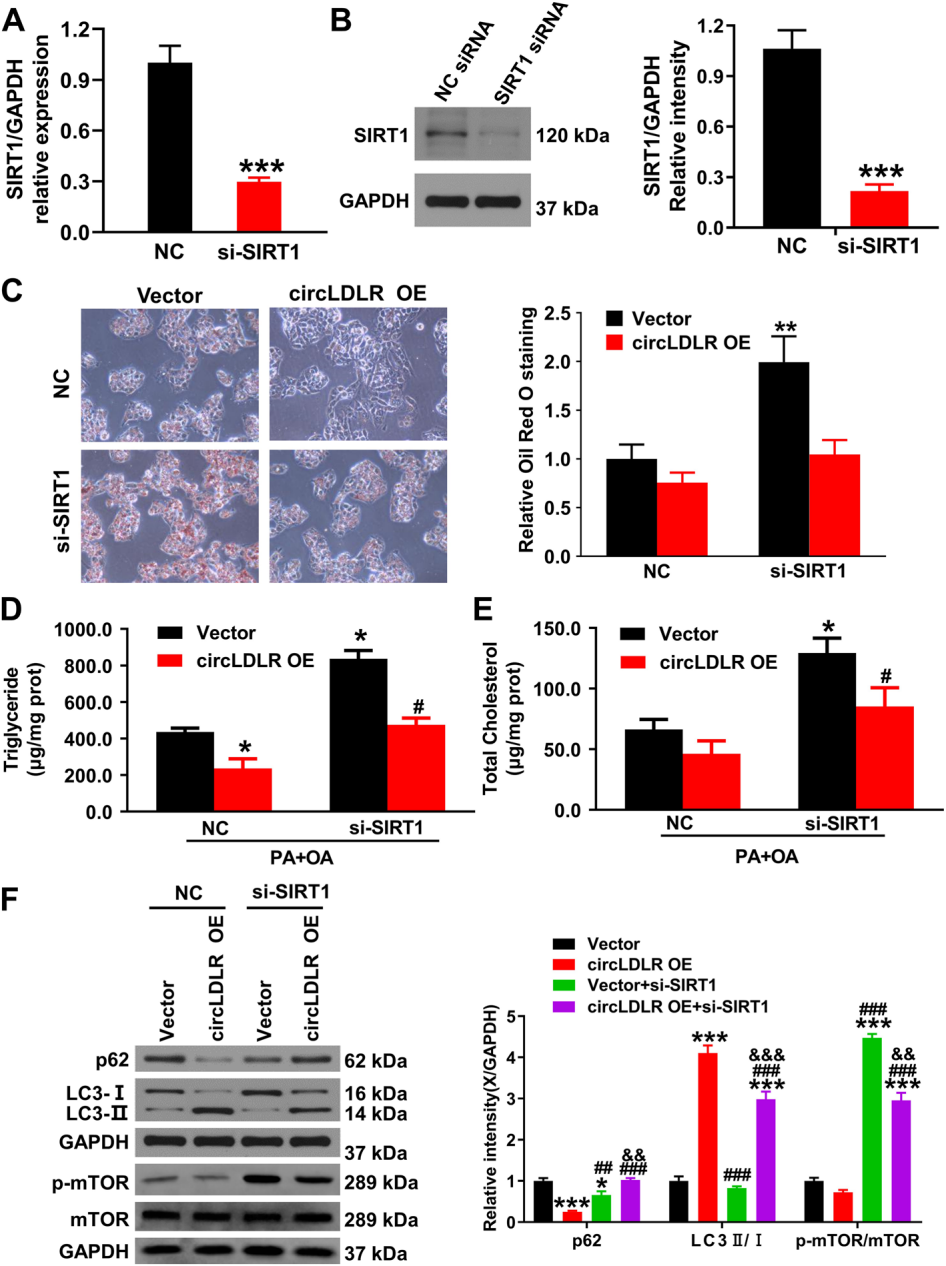
circLDLR regulates the SIRT1-autophagy signaling pathway to alleviate cells’ lipid accumulation. **A** The SIRT1 mRNA expression in Hepa1-6 cells. ^***^P < 0.001 *vs.* NC siRNA group. **B** The SIRT1 protein level in Hepa1-6 cells was determined using western blot. ^***^P < 0.001 *vs.* NC siRNA group. **C** ORO staining was determined to study the lipogenesis of si-SIRT1 in Hepa1-6 cells. ^**^P < 0.01 *vs.* NC group. **D** and **E** The TG and TC contents in Hepa1-6 cells were detected via an enzymatic method. ^*^P < 0.05 *vs.* NC group; ^#^*P* < 0.05 *vs.* vector group. **F** LC3 and p62 expressions in Hepa1-6 cells were determined through western blot and relative quantification. ^*^P < 0.05, ^***^P < 0.001 *vs.* vector group; ^##^*P* < 0.01, ^###^*P* < 0.001 *vs.* circLDLR OE group; ^&&^*P* < 0.01, ^&&&^*P* < 0.001 *vs.* vector + si-SIRT1 group

To figure out the circLDLR’s underlying mechanism in regulating the autophagy pathway, the present study knocked down SIRT1 in Hepa1-6 cells. Results from qRT-PCR and western blotting indicated that knocking out SIRT1 induced a significant diminishment in the SIRT1 protein and mRNA expression level (Fig. 6A and B, *P* < 0.001). Also, ORO staining results showed that SIRT1 knockdown could counteract the inhibition of circLDLR on lipids (Fig. 6C), and the results of TG (Fig. 6D) and TC (Fig. 6E) were consistent with the above, suggesting that the effect of circRNA on cellular lipid metabolism depends on SIRT1. Furthermore, the phosphorylation level of mTOR was significantly higher when circSIRT1 was knocked down compared to the NC group. The circLDLR OE + si-SIRT1 group had remarkably lower LC3-II levels than the circLDLR group (*P* < 0.001, Fig. 6F), while having significantly higher ones than the vector + si-SIRT1 group (*P* < 0.001). The vector + si-SIRT1 group and the circLDLR OE + si-SIRT1 group (*P* < 0.001) had prominently higher levels of p62 than the circLDLR OE group. The above results also indicated that SIRT1 knockdown could counteract the inhibitory impact of circLDLR on p62 and LC3II/I. Together, the inhibition of cellular lipid deposition following circLDLR overexpression was dependent on the regulation of autophagy by SIRT1, while SIRT1 knockdown promoted NAFLD development.

## Discussion

NAFLD has become a globally prevalent chronic disease, which represents a huge hazard to human health. In the present study, excess lipid deposition in cells was effectively rescued by transfection of overexpressed circLDLR, which may be attributed to the downregulation of p62 and upregulation of LC3. At the same time, miR-667-5p overexpression promoted lipid accumulation, TG and TC secretion in NAFLD in vitro. As a mechanism, this study discovered that miR-667-5p directly targets SIRT1 and inhibited the autophagy signaling pathway, while circLDLR acts as a miR-667-5p sponge in activating the autophagy signaling pathway.

CircRNAs with circular structure shows high stability and contains multiple microRNA binding sites [27]. Furthermore, some circRNAs are associated with NAFLD and can serve as competing endogenous RNAs (ceRNAs) via sponge miRNAs [28, 29]. Additionally, it has been observed that the circRNA-miRNA-mRNA axis is engaged in various signaling cascades (such as apoptosis, invasion, and metastasis) and regulates the expression of the pathogenicity-related gene at the transcriptional or post-transcriptional level [27, 30]. In our study, circLDLR expression level was prominently lessened in OA/PA-treated Hepa1-6 cells. However, transfection with circLDLR significantly curtailed the lipid accumulation in cells, indicating that circLDLR serves as a directer in regulating the hepatic steatosis development. Meanwhile, the in vivo results demonstrated that *c*ircLDLR significantly up-regulated LC3 and inhibited p62, and 3-MA could reverse the impacts of circLDLR overexpression, indicating that circLDLR overexpression could improve the NAFLD development via the autophagy signaling pathway activation.

NAFLD has been reported with an enormous number of deregulated miRNAs, such as miR-188-3p [14] and miR206 [15]. And it has been demonstrated that miR-667-5p is critical for osteoarthritis [31]. Nevertheless, the potential function of miR-667-5p in NAFLD remains unclear. Herein, this study explored the effect of miR-667-5p in NAFLD and discovered that miR-667-5p mimic could reduce the SIRT1 protein expression level. SIRT1 is a deacetylase depending on NAD^+^, which acts as a major metabolic sensor of NAD, regulates cellular metabolism, and plays an essential role in regulating hepatic lipid metabolism [32, 33]. An earlier study showed that SIRT1 expression was downregulated in liver tissue from a mouse NAFLD model [11]. Meanwhile, SIRT1 has been demonstrated to have a significant biological effect in regulating lipid metabolism, oxidative stress, and inflammation in the liver as fatty liver disease progresses [34]. Interestingly, recent research has suggested that various noncoding RNAs can regulate the expression of SIRT1 [11], however, the association of SIRT1 with miR-667-5p in NAFLD is still unclear.

It has been shown that fully silencing and down-regulating SIRT1 amplifies fatty liver and inflammation [35]. Lee et al. [36] demonstrated that miR-34a in diet-induced obese mice negatively feedback-regulated SIRT1 levels in the liver. In our study, dual-luciferase reporter gene assay identified miR-667-5p as the binding target for circLDLR and SIRT1 as the target for miR-667-5p. Meanwhile, this study also found that miR-667-5p is another target of SIRT1 to diminish the effect of SIRT1-autophagy signaling in NAFLD disease. However, knockdown of SIRT1 increased lipid accumulation and secretion of TG and TC compared with circLDLR overexpression alone. In summary, these findings indicate that there is a potential association between NAFLD and anomalies in circLDLR/miR-667-5p/SIRT1 signaling.

The present study further explored whether the circLDLR/miR-667-5p/SIRT1 axis alleviates hepatosteatosis by regulating autophagy in the liver. A previous study has indicated that autophagy is crucial for the intracellular storage and utilization of lipids [37]. LC3 has been proven as an autophagy marker, and p62/ sequestosome 1(SQSTM1) has been identified as a selective substrate of autophagy as well as a marker of autophagic flux[38]. There is a short interaction region between p62 and LC3, which can lead to the specific degradation of p62 by autophagy [39]. Furthermore, SIRT1 deacetylates LC3 under cell starvation, which is a critical step in the lipidation process [40, 41]. In our study, overexpression of circLDLR greatly boosted the GFP-LC3 and RFP-LC3 puncta colocalization in OA/PA-induced Hepa1-6 cells, indicating that cicrLDLR can significantly increase the activation of autophagy and the formation of autophagic flux. Next, overexpression of circLDLR in NAFLD mice raised the LC3 protein level and reduced the p62 protein level, revealing that overexpressed circLDLR in vivo could activate the autophagy pathway. However, the results in vitro showed that miR-667-5p significantly raised the p62 protein levels and TG and TC contents, which was in line with those obtained after SIRT1 knockdown, further suggesting that miR-667-5p mimic or knockdown of SIRT1 might promote NAFLD by inhibiting autophagic flux. Taken together, circLDLR and SIRT1 are common targets of miR-667-5p and contribute to the development of NALFD by regulating the autophagy pathway.

### Comparisons with other studies and what the current work adds to the existing knowledge

The outcomes of this paper are novel: the miR-667-5p mimic could reduce the protein expression level of SIRT1.

### Study strengths and limitations

The present research has several strengths. It is the first to find the circLDLR/miR-667-5p/SIRT1 axis in NAFLD, providing a new molecular target for NAFLD therapy development. Limitations in this study should be acknowledged. The prevention and control of NAFLD by circLDLR still needs further research.

## Conclusions

**Fig. 7:**
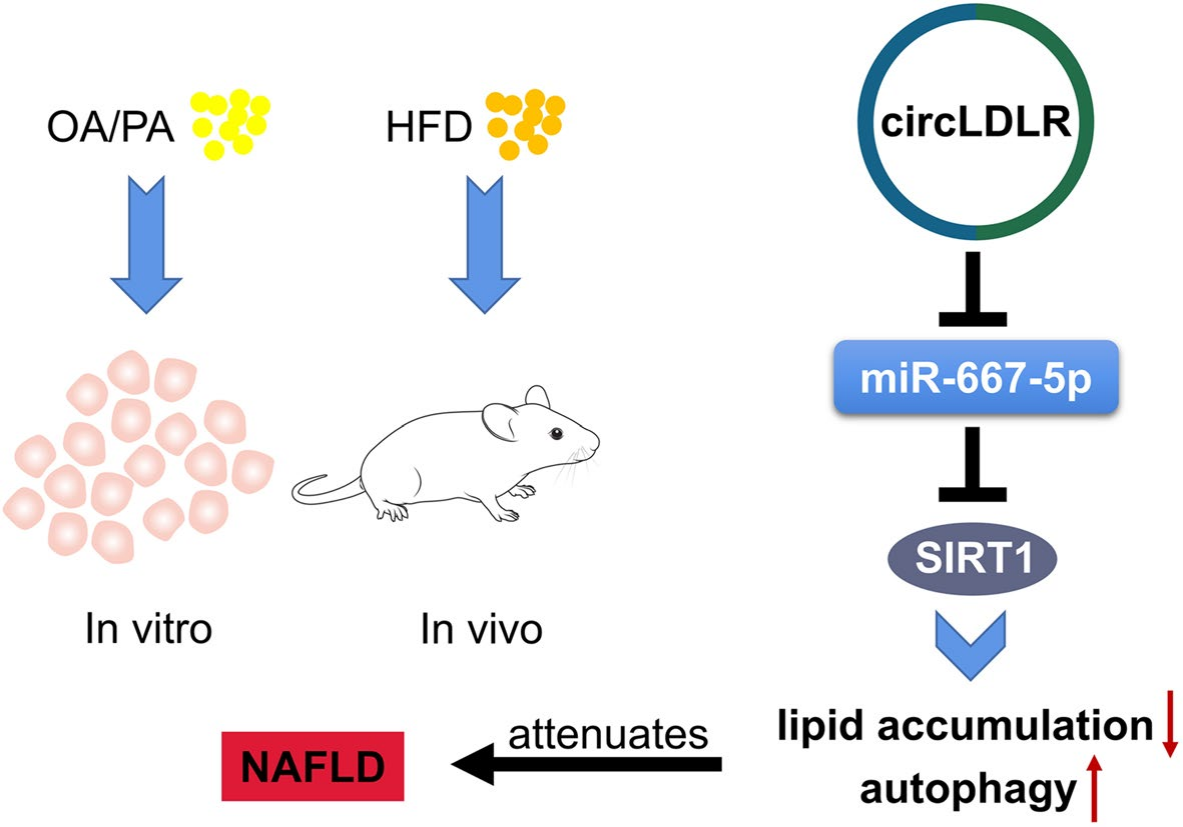
Summary of the study

CircLDLR/miR-667-5p/SIRT1 axis partially attenuates NAFLD through autophagy activation, suggesting that targeting circLDLR could be a promising treatment approach for NAFLD (Fig. 7).

## Data Availability

The data that support the findings of this study are available on request from the corresponding author. The data are not publicly available due to privacy or ethical restrictions.

## Abbreviations

3-MA: 3-Methyladenine
Ad: Adenovirus
AMPK: Adenosine 5 ‘-monophosphate-activated protein kinase
ANOVA: Analysis of variance
BSA: Bovine serum albumin
ceRNAs: Competing endogenous RNAs
circLDLR: Circular RNA low-density lipoprotein receptor
circRNAs: Circular RNAs
DEPC: Paraformaldehyde
DMEM: Dulbecco’s modified Eagle’s medium
ER: Endoplasmic reticulum
FISH: Fluorescence in situ hybridization
GAPDH: Glyceraldehyde 3-phosphate dehydrogenase
GFP: Green fluorescent protein
Hepa1-6: Hepatocytes
HFD: High-fat diet
LC3: Light Chain Microtubule-Associated Protein 3
miRNAs: MicroRNAs
Mut: Mutant
NAD: Nicotinamide adenine dinucleotide
NAFLD: Nonalcoholic fatty liver disease
NC: Negative control
OA: Oleic acid
ORO staining: Oil red O staining
p62: Sequestosome-1
PA: Palmitic acid
PBS: Phosphate Buffered Saline
PFU: Plaque-forming units
PVDF: Polyvinylidene difluoride
qRT-PCR: Quantitative real-time polymerase chain reaction
RFP: Red-Fluorescent Protein
ROCK1: Rho-associated, coiled-coil containing protein kinase 1
SD: Standard deviation
SDS-PAGE: Sodium Dodecyl Sulfate Poly Acrylamide Gel Electrophoresis
SIRT1: Sirtuin 1
snRNA: Small nuclear RNA
SQSTM1: Sequestosome 1
TG: Triglyceride
TC: Total cholesterol
SIRT1 OE: SIRT1 overexpression
Wt: Wild-type
